# Cystinosin, MPDU1, SWEETs and KDELR Belong to a Well-Defined Protein Family with Putative Function of Cargo Receptors Involved in Vesicle Trafficking

**DOI:** 10.1371/journal.pone.0030876

**Published:** 2012-02-17

**Authors:** Vladimir Saudek

**Affiliations:** University of Cambridge Metabolic Research Labs, Institute of Metabolic Science, Addenbrooke's Hospital, Cambridge, United Kingdom; Kings College, London, United Kingdom

## Abstract

Classification of proteins into families based on remote homology often helps prediction of their biological function. Here we describe prediction of protein cargo receptors involved in vesicle formation and protein trafficking. Hidden Markov model profile-to-profile searches in protein databases using endoplasmic reticulum lumen protein retaining receptors (KDEL, Erd2) as query reveal a large and diverse family of proteins with seven transmembrane helices and common topology and, most likely, similar function. Their coding genes exist in all eukaryota and in several prokaryota. Some are responsible for metabolic diseases (cystinosis, congenital disorder of glycosylation), others are candidate genes for genetic disorders (cleft lip and palate, certain forms of cancer) or solute uptake and efflux (SWEETs) and many have not yet been assigned a function. Comparison with the properties of KDEL receptors suggests that the family members could be involved in protein trafficking and serve as cargo receptors. This prediction sheds new light on a range of biologically, medically and agronomically important proteins and could open the way to discovering the function of many genes not yet annotated. Experimental testing is suggested.

## Introduction

One of the best described sorting machineries delivering specialised proteins to their appropriate subcellular locations is the early secretory pathway [1]. The system sends freshly synthesised proteins from the endoplasmic reticulum (ER) to the Golgi apparatus (GA) and returns escaped proteins back to ER. The proteins to be delivered contain sorting recognition signals specific for their receptors that incorporate them into transporting complexes coat proteins II and I (COPII and COPI) and package them in transporting vesicles. Several sorting receptors (or cargo receptors) have been characterised in this system. They are all membrane embedded proteins with transmembrane (TM) helical segments varying in number from a single helix to a multipass helix bundles leaving one terminus in the lumen and the other in the cytoplasm with loops of varying lengths between the TM segments in both the cytoplasm and the lumen.

The sorting proteins with the highest known number of TM helices (7) named Erd2 or KDEL receptors are responsible for the retrograde transport from GA to ER. They package proteins with a KDEL amino acid sequence (or variations thereof) that have escaped from ER to GA and return them as a part of the COPI complex. The difference in pH between ER and GA lumen regulates the dissociation/association of the cargo from its receptor. Human cells contain three closely related Erd receptors (numbered 1 to 3). The predicted receptor topology has been confirmed experimentally [2]. The 7 TM helix bundle presents its N-terminus (a single amino acid) to the lumen and the C-terminus (about 15 amino acids) containing the cargo recognising sequence to the cytoplasm. The connecting loops exposed alternately to cytoplasm and lumen are also short (under 20 amino acids).

**Figure 1:**
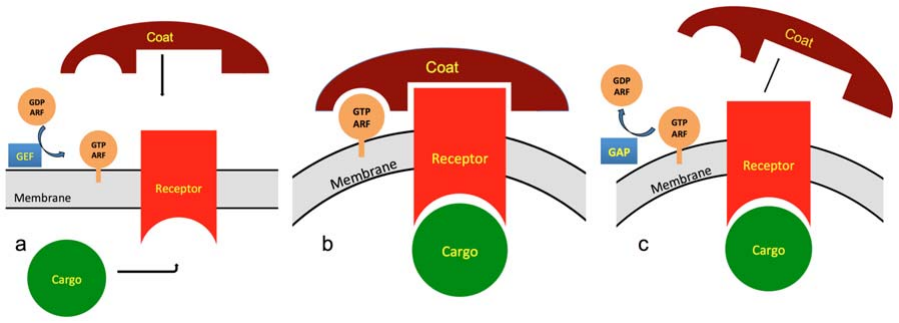
Cargo-vesicle transport. a) assembly and coating, b) coated vesicle membrane, c) uncoating.

The mechanism of cargo-receptor-COPI-vesicle formation is relatively well understood (Fig. 1) [3]. It is regulated by a GTPase ARF1 that cycles between GTP and GDP loaded states. The GTP state is promoted by a guanine exchange factor (GEF). GTP-ARF1 exposes its myristoyl chain which anchors ARF1 to the membrane and recruits the receptor with its cargo and the COPI complex. ARF1 GTPase activity is subsequently promoted by a GTPase activating protein ARFGAP1 and ARF1 returns to its GDP state, sequesters the myristoyl chain and leaves the budding vesicle. Vesicle coating with COPII (anterogade transport from ER to GA) and clathrin (endocytosis) are based on a similar regulatory principle using ARF-like GTPases to control the association of the coat complex with the receptors and membranes [4].

It can be expected that a great variety of cargo receptors may exist to accommodate the diversity of various cargos. In the present report, an extensive search for homologues of KDEL receptors uncovers a large and diverse family named PQ-loop proteins. Discussion of those family members that have been previously investigated experimentally leads to a suggestion that PQ-loop proteins might function as cargo receptors in vesicle transport.

## Results and Discussion

The amino acid sequences of the three aligned human KDEL receptors were used for profile-to-sequence (PSI-BLAST [5], HMMER3 [6]) and profile-to-profile [7] hidden Markov model (HMM) searches. PSI-BLAST identified KDEL receptors in many other species. No additional proteins that could not be annotated as KDEL receptors were found. PSI-BLAST recovered sequences were used for building an HMM profile for HMMER3 searches. No additional significant hit was found. HHpred searches using the aligned sequences of all previous PSI-BLAST hits as query were performed on the profiles from the InterPro collection [8] and on profiles built with proteins from proteomes of several species (human, yeast, *Drosophila melanogaster*, *Caenorhabiditis elegans*, *Arabidopsis thaliana*, yeast and several prokaryota) as implemented in MPI toolkit server [9]. Hits were accepted for further analysis if the E-value was smaller then 10^-5^. In addition, the sequences from the individual proteomes were accepted only if the hit matched all 7 TM helices of the query profile (exceptions discussed below). The HHpred searches were repeated with all human and yeast accepted sequences, with the 3 lowest scoring accepted proteins from the other proteomes and with the InterPro profiles that covered more then just one group of orthologous proteins. The repeated searches provided essentially identical hits as the initial ones, the only difference being the E values.

HHpred searches identified 12 human, 8 *Drosophila melanogaster*, 16 *Caenorhabiditis elegans*, 24 *Arabidopsis thaliana*, 8 yeast and 24 prokaryotic proteins (isoforms and splice variants are not included). The human and yeast proteins are listed in Table 1 and Table 2, (e.g. all human and all yeast proteins in Table 1 and Table 2). the results for other species are available on request. Despite a very conservative inclusion threshold, these collections of sequences are probably exhaustive (final search date November 2011). All other hits received either a much lower E value (2 orders of magnitude) or did not cover all 7 TM helices. Examples of prokaryotic hits are for instance the archeal protein B1L4Q1 (*Korarchaeum cryptofilum*) and the bacterial protein B2JBF6 (*Nostoc punctiforme*).

**Table 1:** Human PQ-loop proteins.

UniProt ID	Swiss-Prot ID	Protein	Gene name	Length
P24390	ERD21_HUMAN	ER lumen protein retaining receptor 1 (KDEL receptor 1)	*KDELR1*	212
P33947	ERD22_HUMAN	ER lumen protein retaining receptor 2 (KDEL receptor 2)	*KDELR2*	212
O43731	ERD23_HUMAN	ER lumen protein retaining receptor 3 (KDEL receptor 3)	*KDELR3*	214
O60931	CTNS_HUMAN	Cystinosin	*CTNS*	367
O75352	MPU1_HUMAN	Mannose-P-dolichol utilization defect 1 protein	*MPDU1*	247
Q9BRV3	SWET1 _HUMAN	Sugar transporter SWEET1, RAG1-activating protein 1 (Stromal cell protein)	*SLC50A1 RAG1AP1*	221
O96005	CLPT1_HUMAN	Cleft lip and palate transmembrane protein 1	*CLPTM1*	669
Q96KA5	CLP1L_HUMAN	Cleft lip and palate transmembrane protein 1-like protein (Cisplatin resistance-related protein 9)	*CLPTM1L*	538
Q2T9K0	TMM44_HUMAN	Transmembrane protein 44	*TMEM44*	475
Q8N2U9	PQLC1_HUMAN	PQ-loop repeat-containing protein 1	*PQLC1*	271
Q6ZP29	PQLC2_HUMAN	PQ-loop repeat-containing protein 2	*PQLC2*	291
Q8N755	PQLC3_HUMAN	PQ-loop repeat-containing protein 3	*PQLC3*	202
O95563*	BR44_HUMAN	Brain protein 44 protein	*BRP44*	127
Q9Y5U8*	BR44L_HUMAN	Brain protein 44-like protein	*BRP44L*	109
A1A4F0*	CC055_HUMAN	Putative uncharacterized protein C3orf55	*C3orf55*	135

*Short sequences homologous to both N- and C-terminal part of the other proteins in the table.

**Table 2:** Yeast PQ-loop proteins.

UniProt ID	Swiss-Prot ID	Protein name	Gene name	Length
Q12010	YO092_YEAST	Uncharacterized membrane protein YOL092W	*YOL092W*	308
Q03193	YD090_YEAST	Uncharacterized membrane protein YDR090C	*YDR090C*	310
P38279	RTC2_YEAST	Restriction of telomere capping protein 2	*RTC2, YBR147W*	296
P17261	ERS1_YEAST	Cystine transporter (ERD suppressor)	*ERS1, YCR075C*	260
Q06328	YD352_YEAST	Vacuolar integral membrane protein YDR352W	*YDR352W*	317
P18414	ERD2_YEAST	ER lumen protein retaining receptor (HDEL receptor)	*ERD2, YBL040C*	219
P25565	YCA2_YEAST	Putative uncharacterized protein YCL002C	*YCL002C, YCL2C*	251
Q03687	YMP0_YEAST	Uncharacterized membrane protein YMR010W	*YMR010W*	405

**Figure 2:**
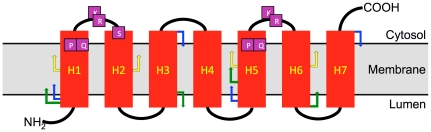
Schematic illustration of the topology of PQ-loop proteins. Helices are shown in red, the termini and connecting loops in black. Arrows indicate the approximate boundaries of the InterPro annotated regions in human proteins: CTNSR (yellow), PQLR (green), MtN3_saliva (blue). The most conserved amino acids are indicated in violet boxes. For the exact sequence positions of these features see Fig. 3 in which the same colour scheme is used.

The searches thus revealed a large, well-defined, self-contained and diverse gene/protein family. Many of its members are described in the curated database Swiss-Prot (e.g. all human and all yeast proteins in Table 1 and Table 2). They had been therefore subjected to an expert annotation. As several of them are listed as PQ-loop repeat containing (see below), the whole set will be referred to in the present report as PQ-loop family with the individual members PQ-loop proteins/genes. Most are predicted to be membrane proteins with 7 TM helices and a common topology. The prediction had been confirmed experimentally for KDEL receptors [2], Cystinosin [10] and for all yeast proteins [11]. Figure 2 illustrates the common topology of the family.

**Figure 3:**
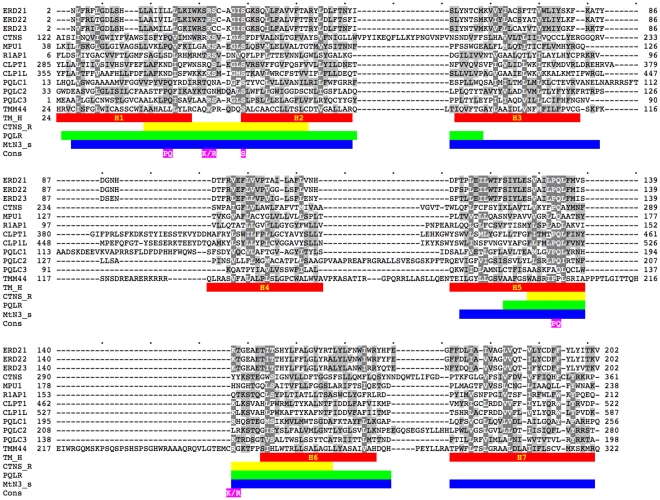
Sequence alignment of human PQ-loop proteins. The non-homologuous N- and C-termini are omitted. Swiss-Prot IDs without the species indication (cf. Table 1) are used. The positions of various sequence features are indicated by coloured bars: Predicted consensus positions of the tramsmembrane helices (TM_H) are indicated in red. Position of the InterPro annotations for Cystinosin, PQL1 and R1AP1 respectively: Cystinosin repeat (CTNSR, yellow), PQ-loop repeat (PQLR, green), MtN3_saliva (MtN3_s, blue). The positions of highly conserved amino acids are highlighted in violet.

Sequence alignment (Fig. 3 shows human proteins as an example) allows the position and the number of TM helices (red in Fig. 3) to be established more precisely then for individual sequences. It also delineates the lengths and position of the non-membrane loops and N- and C-terminal domains. For instance, the positions and number of the helices in PQL1, 2 and 3 proteins were impossible to predict with confidence on individual sequences and no consensus had been reached in the literature for MPDU1. The sequences are very diverse. No amino acid is conserved absolutely and the conservation pattern is essentially invisible to the eye. The most common motif appears to be a doublet of proline and glutamine (PQ, indicated in Figs. 2 and 3). The conservation of the PQ doublet is higher in the C-terminal part is better conserved in the C-terminal part but even here there are exceptions. There are two highly conserved columns with basic amino acids and one with serine. These amino acids (highlighted in Figs. 2 and 3) have yet to be assigned functional properties. The conservation (and the PQ-loop family membership) resides almost exclusively in the helical TM part and the loops between helices 1–2 and 5–6. Many PQ-loop orthologues have large N-terminal and C-terminal extra-membrane domains and extended loops between helices, which are specific only to them and show no homology to other PQ-loop paralogues. They are however well conserved in the orthologues and thus indicate potential functional regions.

HHpred searches also recognised several profiles from the InterPro collection [8]: PQ-loop, MtN3_slv, CTNS, UPF0041, ER_lumen_receptor, pthr12226, pthr10585, PIRSF023381, pthr21347, TIGR00951. The first three are partially overlapping profiles that the query usually hits twice (Figs. 2 and 3). They are indeed annotated as repeats and had been developed independently using the most conserved parts of the seed proteins. The PQ-loop profile spans two TM helices with their connecting loop being the best conserved part of the region. It often contains the amino acid doublet PQ, hence the name [12]. MtN3_slv encompasses a very similar region built initially with the sequences expressed in salivary glands in Drosophila embryo and also contains many sequences of proteins expressed in developing nodules of plants. CTNS SMART [13] profile is the shortest profile (∼30 amino acids) with the broadest specificity covering the tips of two helices and the connecting loop with the frequent PQ doublet. It had been developed by searching databases using the most conserved region of cystinosin. All these InterPro features are schematically indicated on the topology scheme (Fig. 2) and the alignment (Fig. 3). None recognises all PQ-loop proteins. In contrast, the present searches recognise all InterPro CTNSR, PQLR and MtN3_slv annotated proteins. The InterPro descriptions do not contribute to the functional annotation of the family members. In fact, the annotations in the InterPro database are rather misleading; they only mechanically transfer some arbitrarily chosen properties of the better annotated family members to the whole family (e. g. lipid A Biosynthesis N-terminal domain, drosophila saliva domain, sequence involved in root nodule development).

The remaining InterPro profiles had been constructed using alignments of orthologous proteins. They are therefore narrower in specificity and cover nearly the whole sequence including the non-helical parts. They are consequently specific for subsets of the sequences recognised in the individual proteomes and confirm the HHpred searches performed in the present report. For instance ER_lumen_receptor profile had been developed for KDEL receptors and recognises human and yeast ERD2 sequences from Table 1 and Table 2 as well as their *C.elegans* and *D.melanogaster* orthologues.

HHpred searches also provided many significant hits over just half of the query (3 TM helices). Most were alternative alignments of the proteins already identified or even of the query itself. It appears that PQ-loop proteins display a very strong profile homology between their N- and C-terminal parts delineated by the 3 N- and the 3 C-terminal helices. This is an extension of the previous observation that the PQ-loop sequences contain two repeats; the duplication in fact encompasses the whole helical body including the interleaving loops. The finding strengthens the suggestion [12] that PQ-loop genes evolved through gene duplication. The profound divergence of the N- and C- termini indicates that this event may have happened early in their evolution, probably before the divergence of the whole family. Alternatively, PQ-loop genes may have emerged by gene fusion of two shorter homologous but already diverged sequences. Such short proteins do indeed exist: the searches provided very significant hits (E<10^−16^) to proteins shorter then a typical PQ-loop protein whose sequences covered just half of the query and in which 3 TM helices could be recognised. The human proteins are listed as the last three entries in Table 1. Many further hits in other species included eukaryotic proteins as well as the InterPro profile UPF0041 (developed for proteins of unknown function). In the case of prokaryota (e.g. archeal A9A2D4 or Q8PVK4 and bacterial B2IZ35 or Q04MF2), these hits were more frequent than the hits to proteins covering all 7TM part. These shorter proteins could fold in a similar 7TM membrane structure and exercise a similar function as the PQ-loop proteins if they formed dimers or heterodimers. Their inclusion in the PQ-loop family can be justified if formation of dimers is established experimentally.

**Figure 4:**
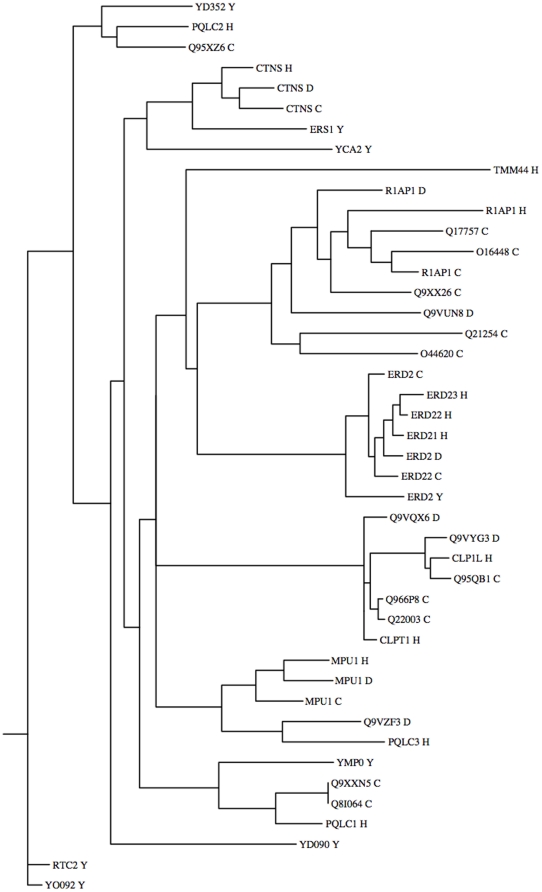
Phylogenic tree of PQ-loop proteins from four animal proteomes. UniProt/Swiss-Prot IDs are used with species identification H, Y, C and D for human, yeast, *C.elegans* and *D.melanogaster* respectively.

PQ-loop genes must have emerged early in evolution. They can be found in all eukaryota. Prokaryota also possess them but not as a rule. *ERD2*, *CTNS*, *MPDU1* and *RAG1AP1/SWEET1* have well-established orthologues in many eukaryota including plants. The phylogenic tree of proteins from human and model organisms proteins (Fig. 4) indicates that the majority of human genes have clear orthologues (e.g. CTNS, MPDU1, CLPTM1 and CLPTM1L). Some genes expanded in just certain organisms, e.g. one chordata gene RAG1AP1 expanded in 17 SWEET orthologues in *Arabidopsis*, 22 in rice, 7 in *C.elegans* and 2 in *D.melanogaster*. TMEM44 appears to be the youngest gene present only in the vertebrate genomes.

Many of the PQ-loop genes had been grouped together previously at least partially. Pfam database [14] lists them as a clan. Some were identified while searching for distant homologues of bacterial rhodopsins [15]. *CTNS* [16] and *MPDU1* [17] are associated with an important phenotype and their impairment leads to serious genetic diseases that prompted intense research. Sequence variants in the proximity of *CLPTM1L* are associated with the risk of many types of cancer [18]. *CLPTM1* is a candidate gene for cleft lip with or without cleft palate malformation [19], [20]. KDEL receptors apart, the molecular mechanism of their function is not clear even for the most studied ones (cystinosin, MPDU1). The function of most of the others remains to be discovered. The largest group, plant PQ-loop set of genes *SWEET*s or nodulin *MtN3* [21], remained enigmatic until they were hit recently in a high throughput search for a specific physiological function [22]. The search for 7TM proteins in prokaryota missed them completely [23].

### Putative function of PQ-loop proteins

The following brief review of the functional properties of the experimentally investigated PQ-loop proteins examines the possibility that their function is related to protein trafficking. Nothing is known about the prokaryotic PQ-loop proteins, however vesicle formation in prokaryota exists [24] and an evolutionary link between vesicle formation in eukaryota and prokaryota has been established [25].

PQ-loop genes have never been considered, to the best of our knowledge, as being functionally related. Their common 7TM structure and topology and homology to the well-known protein trafficking receptors strongly suggest that they might all be cargo receptors. The subcellular location of several PQ-loop proteins is known from high throughput studies and/or focussed experiments and compiled in the Swiss-Prot database. They are found predominantly in membranes of organelles such as ER and GA (ERD2, MPDU1, R1AP1), mitochondria (yeast RTC2), lysosomes (cystinosins), vacuoles (yeast ERS1 and YO092), chloroplasts (*A.thaliana* Q9C9M9) and not in the cell membrane. Preliminary unpublished results indicate that TMEM44 is located in ER and probably also in cytoplasmic vesicles (personal communication, Robert Semple). The location of the proteins is consistent with their putative sorting function. Grouping them together based on their common structural features and evolutionary relationship may help to cast light on their function and help design new experiments.

#### Cystinosin

Cystinosin is a key protein required for clearing cystine from lysosomes. Its point mutational defects cause cystinosis, a recessive autosomal disorder of cystine clearance leading to renal failure [16]. It is generally thought that cystinosin is a cystine transporter. It has been shown that when cystinosin was forced to insert into the cytoplasmic membrane, it enabled cystine transport [26]. The dependence of its function on pH suggested that it was an H^+^ driven sympoter coupling its function with the vacuolar (H+)-ATPase [26]. The sequence however bears no similarity to any known transporter and no associated ATPase has been reported. Yeast contains a homologue Ers1 from the PQ-loop family (Table 2). Human cystinosin and yeast Ers1 are mutual first BLAST hits and, as seen from Fig. 4, they might be functional orthologues. To date there is no evidence that Ers1 facilitates cystine transport. Although Ers1 is not a vital protein under normal conditions, its loss of function results in sensitivity to an antibiotic Hygromycin B [27]. Mutants with deleted *Ers1* can be rescued with human *CTNS*. Ers1 localises in the vacuole (yeast equivalent of lysosome) and in the punctate pattern (endosome), the compartment transporting molecules from outside the cell membrane to the vacuole. A high copy suppressor of deleted *Ers1*, protein Meh1 (alias EGO1), that interacts with a GTPase Gtr1 was identified [27]. These two proteins have been shown subsequently [28], [29] to be members of a multiprotein complex (named Ego/Gse, reviewed in a more general functional context in [30], sorting its cargo from membrane to lysosome. The complex is reminiscent of the vesicle protein coats (Fig. 1) COPI and COPII sorting proteins between ER and GA. In such a system, Ers1 and Cystinosin would not serve directly as membrane transporters but rather as receptor proteins bringing their cargo to lysosome via endosome. Preliminary results [31] indicate that cystinosin has a role to play in the vesicular trafficking and membrane fusion. Cystinosin is not confined just to the lysosomes;it has been observed to move from phagosome to lysosome in *C.elegans* [32]. It is worth noting that *Ers1* was originally cloned as *Erd1* suppressor [33]. Erd proteins 1 and 2 were the first ER retention receptors for ER lumenal soluble proteins in yeast [34] (7 TM KDEL receptors - HDEL in yeast - are from the Erd2 family). The difference in pH between the endosome and the lysosome would be responsible for the dissociation of the cargo from its receptor as in, for instance, HDEL receptors. Interestingly, the rescue function of cystinosin in yeast worked with truncated cystinosin, lacking the 120 amino acid lumenal N-terminus. Ers1 contains only a very short lumenal portion. It is conceivable that cystinosin developed into a specialized receptor from a general cargo receptor such as Ers1. As the known cargo receptors carry proteins rather then metabolites, it would seem probable that cystinosin carries a transporter rather then cystine itself. Indeed, the Ego/Gse complex sorts amino acid transporters into lysosomes [29].

Cystinosis patients develop Fanconi syndrome caused by defective reabsorption of various solutes by the renal proximal tubule, e.g. phosphate, bicarbonate, amino acids and glucose [35]. The pathology is not suppressed by the cystinosis drug cysteamine that only facilitates clearance of cystine from lysosomes [35]. It has been demonstrated recently [36] that proximal tubule cells in which the Cystinosin gene has been knocked down show significant reduction of the major Na^+^/Pi contransporters in the apical membrane. It is tempting to suggest that Fanconi syndrome in cystinosis could be due to the defective sorting of various solute contransporters to their correct place of action because of the defective sorting receptor Cystinosin.

#### Mannose-P-dolichol utilization defect 1 protein

The function of MPDU1 is unknown. It is needed in all known classes of monosaccharide-P-dolichol-dependent glycosyltransferase reactions in mammals, i.e. mannosylation and glucosylation of lipid-linked oligosaccharides, the mannosylation of glycosylphosphatidylinositols, the *C*-mannosylation of tryptophanyl residues, and protein *O*-mannosylation [37]. Mutations in this gene are the cause of congenital disorder of glycosylation (CDG) type 1F, a severe disease linked to defects in protein N-glycosylation [17]. The growth of the oligosaccharide chain before it is transferred to the right protein depends on the stepwise action of 17 well-described membrane-embedded glycosyl transferases. The mutation of any of them can lead to CDG and patients with a mutation in 13 of them have been observed. *MPDU1* and *RFT1* are two additional genes behind CDG whose function is not clear. The growing oligosaccharide chain is attached to the ER membrane by a lipid carrier, dolichol. The synthesis is carried out first outside and subsequently inside ER. Dolichol needs therefore to be flipped to expose the oligosaccharide chain to the lumen. RFT1 is proposed to be the missing flippase that is needed to transfer the growing chain from outside to inside ER [38] but this notion is not accepted generally [39]. An unpublished bioinformatics analysis outside the scope of the present paper but applying the same methods shows that RFT1 is in fact a 12 TM transporter related to multidrug and toxin extrusion proteins. Other known flippases are indeed lipid transporters [40]. A recent paper shows that there are actually at least three different flippases in ER responsible for flipping dolichol-linked saccharides [41]. MPDU1 could thus be the sorting carrier bringing/retaining these flippases (and perhaps other proteins) in ER.

#### RAG1-activating protein 1, RAG1AP1

RAG1AP1 (recently renamed SWEET1 [22]) regulates the trafficking of an ion channel TRPV2 (responsible for extreme temperature sensing) from the perinuclear structures (ER or GA) to the cell membrane [42] in mammalian cells. TRPV2 and RAG1AP1 associate physically during protein synthesis and regulate TRPV2 cell-surface presentation but RAG1AP1 itself never appears in the cellular membrane. Overexpression of RAG1AP1 increases cell surface levels of TRPV2 significantly. Although not recognised as a PQ-loop protein or even as a 7TM protein, it has been shown to be a specific protein receptor transporter of TRPV2 in vesicles from the place of its synthesis to the cell membrane.

#### SWEETs

Several members of a large group of plant PQ-loop proteins named SWEETs (at least 6 out of 17 in Arabidopsis, 2 out of over 20 in rice) when overexpressed in human or yeast cells support cell membrane uptake and ER efflux of sugars [22]. Similarly, as for cystine clearance by Cystinosin [26], the simplest explanation is that SWEETs are sugar transporters. They were therefore assigned the function of bidirectional uniporters (facilitators, solute carriers) [22]. Their membership in the PQ-loop family leads however to an alternative interpretation of the experimental data: SWEETs interact with sugar (and possibly other) transporters and carry them to the right compartment, i.e. they function as cargo receptors trafficking transporters to their site of function. Indeed, the only mammalian orthologue of plant SWEETs, RAG1AP1 (alias SWEET1), has been shown experimentally to be an ion channel carrier ([42], see above).

#### Testing whether PQ-loop proteins are cargo receptors participating in vesicle trafficking

Determining the organelle location of PQ-loop proteins would be the first step towards identifying their function. They could be identified in two or more different compartments. As sorting depends crucially on ARF-like, GTPase activating protein-like and guanine exchange factor-like proteins (Fig. 1), useful tools would be nonhydrolysable GTP analogues (e.g. Guanosine gamma thio-phosphate, 5′-Guanylyl imidodiphosphate), which lock activated ARF on the membrane and the antibiotic Brefeldin A, which inhibits activation and thus recruitment of the vesicle coating complex to the membrane. There are at least 32 genes for ARF proteins and more than 135 related GTPases in the human genome and few have been assigned a function. The position of the orthologue specific loops and C- and N-terminal domains can be predicted (Fig. 2) and their ablation or mutation can give useful clues for the function. It should also be possible to identify the cargo(s) in protein-protein interaction studies.

### Conclusions

PQ-loop proteins, as members of one family, are likely to exercise similar function. The protein cargo function is firmly established for KDELR and RAG1AP1/SWEET1. The review of the literature indicates that the experimental data obtained for Cystinosin, MPDU1 and plant SWEETs are consistent with the sorting function of these proteins although they are currently annotated as metabolite (or solute) carriers. Experiments to test their participation in vesicle trafficking could be designed. If positive, identification of their cargo could throw new light on two debilitating congenital diseases. The phenotype of several genes in Table 1 indicates that PQ-loop proteins in general are responsible for important functions. Unravelling their common molecular properties and mode of action could bring important theoretical, practical and medicinal progress.

## Materials and Methods

Sequences were extracted from UniProt database and are labelled with their UniProt or Swiss-Prot IDs. Only those absent from UniProt are from NR protein NCBI database and are labelled with their NCBI IDs. Gene names are printed in italics and protein names in plain text. Homology searches were performed by PSI-BLAST [5] (inclusion threshold E<0.001) and HMMer3 [6] on both UniProt and NR protein NCBI databases. Profile-to-profile searches were conducted using HHPred algorithm [7] as implemented in MPItoolkit [9]. Sequences with amino acid identity higher then 40% were aligned with T-coffee [43]; those with lower homology were aligned using profile-to-profile methods PROMALS [44] and Psi-coffee [45]. Minor differences in the multiple sequence alignments obtained with PROMALS, Psi-coffee and PSI-BLAST were reconciled using the consistency method of T-coffee software. The multiple sequence alignments were analysed and displayed with GeneDoc [46]. Secondary structure was predicted using the consensus method Jpred 3 [47]. TM helices and their topology were predicted combining HMM profiles and homology using PolyPhobius [48]. The phylogenic relationship was analysed using the Phylogeny.fr suite with the approximate likelihood ratio test for branches set to SH-like [49].
